# Conducting ethical internet-based research with vulnerable populations: a qualitative study of bereaved participants’ experiences of online questionnaires

**DOI:** 10.1080/20008198.2018.1506231

**Published:** 2018-08-21

**Authors:** Kirsten V. Smith, Graham R. Thew, Belinda Graham

**Affiliations:** a Oxford Centre for Anxiety Disorders and Trauma, University of Oxford, Oxford, UK; b The Loss Foundation [Registered Charity 1147362], London, UK

**Keywords:** Assessment, grief, ethics, questionnaires, measurement reactivity, internet, Evaluación, Duelo, Ética, Cuestionarios, Reacción a la medición, internet, • Emails analyzed in the present study suggested that conducting grief research online is acceptable to bereaved individuals and suggest it should not be discouraged based on ethical concerns.• We implemented a practical framework for ethical online data collection designed to support participant well-being and monitor risk with bereaved people and other potentially vulnerable populations.• Given the reported benefits of participation, clinical researchers should consider potential measurement reactivity effects when designing longitudinal studies.

## Abstract

**Background**: Bereavement can be considered a potentially traumatic experience, and concerns have been raised about conducting grief research responsibly online.

**Objective**: Given that online research introduces new methodological opportunities and challenges, we aimed to develop a greater understanding of how bereaved individuals experience participation in online research.

**Method**: One day after participation in an online grief study, 876 participants, bereaved on average for 40 months, received a ‘check-in’ email to support well-being and offer further contact if needed. Although not explicitly asked to respond if no help was needed, 300 participants sent email replies, with only six requesting support. These responses were analysed qualitatively using content analysis.

**Results**: Results suggested that participants found it acceptable to be asked about their grief and while difficult emotions were frequently described in response to the questionnaires, these reactions were temporary. A range of positive reactions was also reported, including new realizations arising from completing the research and changes in thinking related to grief. Participants also wrote about their appreciation for the study and how it was carried out, as well as a desire to contribute more to the study and to help others in a similar position.

**Conclusions**: We suggest that the use of the check-in email to support well-being following study completion, along with advice on preparing to take part, contributed to positive experiences of participation and we recommend these strategies for future studies. These findings could allay clinical concerns about conducting online research with vulnerable populations, as well as raising questions about the possible therapeutic impact of measurement.

## Abstract (es)

**Antecedentes**: El duelo se puede considerar una experiencia potencialmente traumática, y han surgido preocupaciones respecto a la realización responsable de investigaciones de duelo en línea.

**Objetivo**: Dado que la investigación en línea plantea nuevas oportunidades y desafíos metodológicos, intentamos ampliar la comprensión sobre cómo las personas en duelo experimentan la participación en investigación en línea.

**Método:** Un día después de participar en un estudio de duelo en línea, 876 participantes en duelo, con un promedio de pérdida de 40 meses, recibieron un correo electrónico de ‘registro’ para apoyar el bienestar y ofrecer contacto adicional de ser necesario. Aunque no se les pidió explícitamente a los participantes que respondieran si no necesitaban ayuda, 300 enviaron respuestas por correo electrónico, con sólo seis de ellos solicitando apoyo. Estas respuestas se analizaron cualitativamente mediante análisis de contenido.

**Resultados:** Los resultados sugirieron que los participantes consideraron aceptable que se les preguntara sobre su dolor y, aunque frecuentemente se describieron emociones difíciles en respuesta a los cuestionarios, estas reacciones fueron temporales. También se reportaron una serie de reacciones positivas, incluyendo nuevos entendimientos derivados de la finalización de la investigación y cambios en el pensamiento relacionado con el duelo. Los participantes también escribieron acerca de su apreciación por el estudio y su ejecución, así como el deseo de contribuir más al estudio y ayudar a otros en una posición similar.

**Conclusiones:** Sugerimos que el uso del correo electrónico de registro para apoyar el bienestar seguido de la participación en el estudio, junto con consejos sobre cómo prepararse para participar, contribuyó a experiencias positivas de participación, y recomendamos el uso de estas estrategias para estudios futuros. Estos hallazgos podrían disipar las preocupaciones clínicas sobre la realización de investigaciones en línea con poblaciones vulnerables, así como plantear preguntas sobre el posible impacto terapéutico de las mediciones.

## 在易感人群中进行有伦理的互联网研究：对丧亲者经验的定性在线问卷研究

**背景：**丧亲被视为潜在的创伤经历，开始有人对在网络上进行哀伤研究是否是负责任的行为表示担忧。

**目标：**在线研究带来了方法上新的机遇和挑战，我们的目的是更好地理解丧亲者对参与在线研究有何体验。

**方法：**876名参与者(平均失去亲人40个月）在参与在线哀伤研究的第二天，收到一封“注册”电子邮件，对其心理状况提供支持，并可在必要时进一步联系。虽然没有明确要求在无需帮助的情况下回复，300名参与者还是发送了电子邮件回复，其中只有6个请求帮助。使用内容分析对这些回复进行定性分析。

**结果：**结果表明，被试表示可以接受对哀伤的提问，虽然在回答问卷时经常描述到痛苦感受，但只是暂时的。被试还报告了一系列积极反应，包括完成问卷时产生的新感想以及对哀伤的看法改变。参与者还提到了他们对该研究及其实施方式的感激，希望为研究做出更多贡献并帮助其他处境相似者的愿望。

**结论：**我们建议使用“注册”电子邮件对研究完成后被试的心理健康提供支持，提供关于如何对参加研究进行准备的建议，并有助于增加积极的参与经验。我们建议未来的研究可以使用这些策略。这些发现可以消解临床上对在线研究弱势群体的担忧，以及指出测量本身可能具有治疗效果的问题。

**关键词：**评估;哀伤;伦理;问卷调查;测量反应性;互联网

## 1. Introduction

The loss of a significant other can be characterized as a potentially traumatic experience that may give rise to mental and physical health consequences. As such, bereaved individuals have been classified as a vulnerable group (Koffman, Morgan, Edmonds, Speck, & Higginson, 2009; Steeves, Kahn, Ropka, & Wise, 2001), raising concerns among those overseeing research studies that asking questions about loss might elevate distress and introduce risks that outweigh the benefits of research participation (see Dyregrov, 2004; Kreicbergs, Valdimarsdóttir, Steineck, & Henter, 2004). Despite this concern, there is growing evidence that bereaved individuals find participating in research a useful experience with the potential to benefit others in similar situations (Beck & Konnert, 2007; Cook & Bosley, 1995; Koffman et al., 2012), and guidelines have been developed to support this process (e.g. Parkes, 1995).

In an important study, Dyregrov (2004) examined bereaved parents’ experiences of participating in research about their grief and found that while 73% reported finding the interviews painful, none regretted participating and 100% described the experience as positive. Participants from this study provided recommendations for grief researchers such as making initial contact remotely (by post), providing them with thorough written information, allowing them to decide the location of the research, giving them adequate time, offering follow-up, and allowing them to give feedback. Despite these recommendations, it is unclear whether, and how often, potentially vulnerable research participants are routinely followed up to support their well-being.

Alongside ethical considerations about the appropriateness of research with bereaved people, it is also important to consider the extent to which research samples are representative of bereaved individuals in general. Stroebe, Stroebe, and Schut (2003) commented on the biases introduced when recruiting participants in religious settings, through hospital death records, and via bereavement support groups. However, recent advances in online data collection and targeted social media advertising have meant that individuals who are not religious, whose loved one did not die in hospital, and who never sought external support in their bereavement can have the same chance of participating in grief research as individuals in the settings mentioned by Stroebe and colleagues. In addition, the internet may prove an uncontroversial arena for participant recruitment because it eliminates the potential for coercion and maximizes participant choice regarding the time, location, and duration of participation, factors that have been shown to be important in interview studies with bereaved people (Bentley & O’Connor, 2015). However, remote data collection brings with it a new set of ethical challenges for researchers to manage. Asking sensitive questions to potentially vulnerable individuals at a distance leaves the researcher with more responsibility to communicate clearly the risks of participation while also attending to any emotional needs that may be generated as a consequence of the research. These concerns are especially important when working with groups previously considered to be particularly at risk, such as the newly bereaved (Rohertson, Jay, & Welch, 1997; Rosenblatt, 1995), bereaved parents (Hynson, Aroni, Bauld, & Sawyer, 2006), and those bereaved by violent means (Jorm, Kelly, & Morgan, 2007).

A recent study (Smith & Ehlers, in preparation) used online questionnaires to assess grief and mental health outcomes related to bereavement. As part of this, all participants were sent an email to check on their well-being a day after completing the questionnaires. Initial reading of participants’ replies to that message suggested that many contained interesting insights on grief and their experiences of completing the questionnaires. These responses were unexpected given that they were unprompted. Therefore, the present study sought to capitalize on the data obtained through these emails in order to understand the impact of online assessment among vulnerable populations.

Specifically, our questions were: (1) To what extent and in what ways do participants choose to respond to an email checking on their well-being after completing online questionnaires about grief? (2) How do those who reply describe their experience of completing these questionnaires? (3) Do they report any positive or negative consequences of the questionnaires?

## 2. Method

### 2.1. Participants

Participants were 876 individuals aged over 18 years who had experienced a bereavement. Participants were recruited through bereavement charity mailing lists, via social media advertisements, and through the Google content network. No restrictions were placed on the length of time since bereavement (months since loss: *M* = 39.71, *SD* = 69.07, *Mdn* = 14.00). Participants were included in the study if they indicated that the deceased represented someone with whom they had a close relationship, as opposed to an acquaintance or a distant friend or relative (length of relationship in months: *M* = 360.43, *SD* = 182.13, *Mdn* = 359.00). Of the 876 participants who completed the online study, 300 replied to the ‘check-in’ email sent 24 hours following completion and were therefore included in this study.

### 2.2. Procedure

The Oxford Grief Study is a questionnaire study investigating the cognitive factors associated with psychological distress following bereavement. Questionnaires covered concepts such as negative appraisals, coping strategies employed when grieving, and loss-related memory characteristics, as well as measures of psychological distress such as depression, posttraumatic stress disorder, and prolonged grief disorder. All questionnaire data were collected remotely using online data collection software that allows measures to be compiled and distributed via the internet (Qualtrics, 2005). The study was approved by the University of Oxford Medical Sciences Inter-Divisional Research Ethics Committee (MS-IDREC-C1-2015-230, MS-IDREC-C1-2015-231). Participant information sheets were provided and informed consent for the use of all data arising from the study was given by all participants prior to taking part. Additional consent was obtained from those participants whose email responses are quoted.

As a result of suggestions from bereaved community members in a patient and public involvement consultation in the development phase of the research, participants who completed the measures received a check-in email from the lead researcher the day after completing the questionnaires. These emails offered further support if needed by providing the opportunity of a telephone call with the lead researcher (a clinical psychologist) to discuss any aspects they had found distressing. The email also included an expression of appreciation that they had participated, condolence for their loss, and normalization of difficult emotions related to answering questions about grief. The email did not ask for a response if no help was needed and did not ask any further questions about their experiences of loss or grief, or request any feedback on the study process. (The email text is available online as supplementary material.)

### 2.3. Analysis

Email replies were analysed using NVivo (version 11.4) following a conventional content analysis approach (Hsieh & Shannon, 2005), where coding categories were developed and refined from the raw data rather than from existing knowledge or theory. This was appropriate for this data given that the email sent to participants was not asking specific research questions and our primary aim was to describe how people chose to respond. Code and theme development was conducted following the guidance by Braun and Clarke (2006), emphasizing familiarity with the data and the iterative refinement of themes.

Firstly, 20 emails were randomly selected and independently reviewed by all three authors (clinical psychologists), who each generated lists of broad themes arising from the data, which were then discussed. Secondly, an additional set of 40 randomly selected emails were independently coded by all authors to identify any remaining themes, thus ensuring that all content could reasonably be coded under the themes identified. Thirdly, all themes were refined through discussion and operationalized into superordinate and subordinate themes to generate a coding manual. Finally, the total sample of emails was then divided equally between the authors and coded independently using the manual. To assess interrater reliability, 20% of emails were coded by all three raters and reliability was good (kappa range 0.72–0.80) (Landis & Koch, 1977).

## 3. Results

Characteristics of participants who responded or did not respond to the check-in email are presented in Table 1. Responders were significantly older than non-responders but did not differ on any other demographic variables, loss characteristics, or psychopathology symptoms. Responders included 55 people (18%) who were within three months of their loss, 61 people (20%) who had lost a child, and 55 people (38%) who were bereaved through violent means. Of those who responded to the check-in email (*n* = 300), six participants took up the offer of a telephone call to discuss aspects of the study that they found difficult or distressing.

**Table 1.** Non-responder and responder demographic variables, loss characteristics, and psychopathology symptoms.

	Non-responders	Responders	
	(*n *= 576)	(*n* = 300)	Statistics
**Demographics**			
Age (years), *M*(*SD*)	47.22 (12.73)	50.69 (12.50)	*t*(874) = −3.83*
Female, *n* (%)	471 (81.8)	237 (79.3)	*χ^2^* = .366^a^
Highest level of education, *n* (%)			*χ^2^*(6) = .123
No qualifications	22 (3.8)	7 (2.3)	
O levels/GCSEs	70 (12.2)	27 (9.0)	
A levels	64 (11.1)	26 (8.7)	
Professional qualification (e.g. teaching, nursing)	80 (13.9)	50 (16.7)	
NVQ/BTech/apprenticeship	48 (8.3)	39 (13.0)	
University degree	164 (67.2)	80 (26.7)	
Postgraduate degree	127 (22.1)	71 (23.7)	
Ethnicity, *n* (%)			*χ^2^* = .135^a^
Caucasian	552 (96.0)	281 (93.7)	
Non-Caucasian^b^	23 (4.0)	19 (6.3)	
**Loss characteristics**			
Months since loss, *M* (range) *Mdn*	41.71 (0–685) 14.00	35.87 (0–548) 15.50	*t*(874) = 1.25
Relationship of deceased, *n*(%)			*χ^2^*(5) = .612
Spouse/partner	197 (34.2)	104 (34.7)	
Child	93 (16.1)	61 (20.3)	
Sibling	37 (6.4)	16 (5.3)	
Parent	180 (31.3)	90 (30.0)	
Other relative	55 (9.5)	22 (7.3)	
Close non-relative	14 (2.4)	7 (2.3)	
Relationship length (months), *M*(*SD*) *Mdn*	354.18 (179.96) 340.50	372.42 (185.94) 372.00	*t*(874) = −1.41
Cause of death, *n* (%)			*χ^2^* = .249^a^
Non-violent^c^	487 (84.7)	88 (15.3)	
Violent^d^	244 (81.6)	55 (18.4)	
**Psychopathology symptoms**			
Prolonged grief,^e^ *M*(*SD*)	31.76 (10.57)	32.35 (10.32)	*t*(874) = −.785
Depression,^f^ *M*(*SD*)	10.88 (7.55)	10.66 (7.41)	*t*(872) = .414
PTSD,^g^ *M*(*SD*)	29.64 (18.23)	29.30 (18.00)	*t*(871) = .198

^a^Fisher’s exact test (2×2).

^b^Groups combined owing to low cell counts.

^c^E.g. illness.

^d^E.g. accident, suicide, homicide, medical negligence, accidental drug overdose.

^e^Measured using the 13-item Prolonged Grief scale (Prigerson & Maciejewski, 2008), range 11–55.

^f^Measured using the nine-item Patient Health Questionnaire (Kroencke, Spitzer, & Williams, 2001), range 0–27.

^g^Measured using the 20-item Posttraumatic Stress Disorder Checklist for DSM-5 (Weathers et al., 2013), range 0–80.

*p < .001.

The superordinate and subordinate themes are described below and summarized in Table 2.

**Table 2.** Frequencies of superordinate and subordinate themes.

Superordinate theme	Frequency, *n* (%)	Subordinate theme	Frequency, *n* (%)
Emotional impact^a^	215 (72)	Okay	95 (32)
		Temporary negative	69 (23)
		No change	41 (14)
		Positive	6 (2)
		Negative	5 (2)
Appreciation	169 (56)	Appreciation of check-in email	139 (46)
		Pleased to be taking part	42 (14)
		Appreciation of opportunity to think/share	15 (5)
Offering more	146 (49)	Sharing more of story	83 (28)
		Grief reflections	62 (21)
		Offer to help more	59 (20)
Participation	105 (35)	I want to help other people	46 (15)
		Found study interesting	26 (9)
		Experience of study process	24 (8)
		Coping strategy after questionnaires	23 (8)
Realizations	46 (15)	Self-awareness	18 (6)
		Seeing where I am at	17 (6)
		Normalizing	10 (3)
		Noticing progress	8 (3)
Cognitive impact	41 (14)	Reflective processing	22 (7)
		Increased thinking	18 (6)
		Induced dreams	4 (1)
Physical impact	12 (4)	Negative impact	10 (3)
		Positive impact	2 (1)
No reflection included	10 (3)		

Percentages reflect the proportion of the total number of responders (*n* = 300) who included a comment coded under that theme.

^a^Emails that included reflections on emotional impact were only coded under one subordinate theme.

### 3.1. Emotional impact

Participants commonly described the way in which they reacted emotionally to completing the questionnaires, or gave a sense of their emotional well-being following participation.

#### 3.1.1. Okay

Many participants described feeling okay or fine after completing the questionnaires, and did not mention feeling good or bad in any way as a result of participating. For some, this indicated a desire to reassure the researcher of their well-being:

[I] want you to know I am doing ﬁne. (Participant 189)

#### 3.1.2. Temporary negative

Other participants reported feeling at least some negative emotional reaction, but also indicated that this was temporary and they were now coping or doing fine:

Yes, when you reﬂect on old wounds it does leave you feeling low, and I will say tears were shed. I can reassure you that I am now feeling ﬁne! (Participant 173)

#### 3.1.3. No change

Some participants clearly outlined that participating in the study had not raised new emotions for them and that they were feeling the same as before completing the questionnaires. This included a sense that participants may have already been experiencing negative emotion that remained negative and unchanged as a result of the questionnaires:

I’m ok, or at least no worse than before – I’m having a bit of a bad spell anyway. (Participant 7)

#### 3.1.4. Positive

While rare, some participants noted feeling positive as a result of completing the questionnaires, without mentioning any initial negative emotional reaction:

It was actually quite cathartic. (Participant 130)

#### 3.1.5. Negative

A small minority indicated feeling negative emotions after completing the questionnaires and did not indicate whether these feelings had abated:

However, the questions did indeed stir up much pain and some unresolved issues. (Participant 265)

### 3.2. Realizations

A significant theme was related to personal realizations that arose from completing the survey. These described previously absent information gained by participants as a result of completing the study.

#### 3.2.1. Self-awareness

Occasionally, participants reported experiencing realizations about themselves, their emotions, or the circumstances of their loss or grief:

It occurred to me that something I have found quite hard to do since my mother passed away last August is to look at photographs of her. I also have a box of her writings that I cannot yet feel able to look at and read. I mention this only as I didn’t get a chance to mention it during the survey – these obstacles only came apparent to me after. (Participant 92)

#### 3.2.2. Seeing where I am at

Others suggested that participating had helped them to situate or locate themselves within an individual grief process or journey:

[The questionnaire was] an opportunity to reﬂect on where l am now in the process of bereavement. (Participant 106)

#### 3.2.3. Normalizing

While rare, some people indicated that the questionnaires had prompted realizations that their feelings are normal and shared by others in a similar position:

Some of the thoughts and feelings mentioned really resonated with me and it’s reassuring to know that it’s not just me that experiences them, it is most deﬁnitely part of this awful grief process. (Participant 274)

#### 3.2.4. Noticing progress

Some emails included a sense that the questionnaires had helped the participant to recognize a positive change in their grief over time that they may otherwise not have noticed:

It did make me realise how far I’ve come since those early days so that was a positive for me personally. (Participant 106)

### 3.3. Cognitive impact

Some participants noticed changes in their thinking or thought patterns related to their grief that may have arisen as a consequence of completing the questionnaires.

#### 3.3.1. Reflective processing

Some participants expressed a sense that it was beneficial to have reflected on the emotions that arose from completing the questionnaires:

I have actually found reﬂecting on and specifying my feelings helpful in making some sense of the turmoil. (Participant 167)

#### 3.3.2. Increased thinking

Some reported that the questionnaires seemed to lead to an increase in thoughts relating to the deceased, or suggested that something was prompted or reactivated with regard to the loss or its circumstances:

Answering the questions has led me to think more about [Name] and her death today. (Participant 284)

#### 3.3.3. Induced dreams

Four participants described having dreams related to the deceased after completing the questionnaires.

### 3.4. Physical impact

Occasionally, some participants noted a physical impact of the questionnaires. Negative effects included tiredness, poor sleep, and headache, while some participants reported a positive effect of improved sleep.

### 3.5. Offering more

A notable theme was participants using their email response to offer further information, reflections, or practical suggestions that others might find helpful.

#### 3.5.1. Sharing more of story

Some participants shared information regarding the circumstances of the loss and its consequences, or offered additional background information about the deceased:

[Name] had always known I would move house if he went ﬁrst, as he would had it been me, and I followed this through with a move to the seaside which gave me practical things to think about. (Participant 144)

#### 3.5.2. Grief reflections

For some, the email was an opportunity to describe their thoughts, reflections, and experiences of grief itself, or the process of grieving:

We don’t want another day to start. Not another day where they are not with us. You don’t think it will ever change. It doesn’t actually. You just learn to encompass it into your life. You are a different person. It is learning to live with this different person that is difficult. I reckon it took me six years before I joined the human race again, which makes a mockery of the people who asked ‘are you over it’ about two months after she died. (Participant 278)

#### 3.5.3. Offer to help more

Others expressed a willingness to participate further in the research, for example by sharing further information or helping in other ways in order to contribute more:

If there is anything I can offer, even above that which is speciﬁed, to your research, I would be very happy to do so. (Participant 167)

### 3.6. Appreciation

Many participants expressed their appreciation for the opportunity to take part in the study or feeling grateful for the way the study was carried out.

#### 3.6.1. Appreciation of check-in email

Many participants commented specifically on the check-in email, going beyond a purely administrative response (such as ‘thanks for your email’), to acknowledge the benefits they experienced from this follow-up message:

Many thanks for your email. Highly appreciative of your kind and aﬃrming words. (Participant 90)

#### 3.6.2. Pleased to be taking part

Some participants expressed a sense of pride or personal meaning in being able to participate in the study:

I’m really happy to be involved in your valuable research. (Participant 272)

#### 3.6.3. Appreciation of opportunity to think/share

Although rare, some reflected on how the questionnaires had given them a welcome chance to share their thoughts and feelings about the loss, or to think about the deceased personally:

It gave me scope to communicate about [him], something which I have found has been lacking as no one seems to mention his name and it was good to just be able to say things about him. (Participant 71)

### 3.7. Participation

Some participants reflected on what it was like to be part of the study, describing elements such as their motivations for taking part or their personal observations made while completing the questionnaires.

#### 3.7.1. I want to help other people

Some participants described their belief that their taking part would be helpful for others who had lost someone. These comments often implied the idea that grief is poorly understood and that better support services are needed:

I am more than happy to participate in the study if it helps to provide better insight into grief and in some way will lead to better support for those who need it. (Participant 148)

#### 3.7.2. Found study interesting

Some people described how taking part had raised their interest in the research topic or generated an interest in their own reactions to questions about grief:

I found the questionnaire very interesting to ﬁll out and I shall look forward to hearing about the research ﬁndings when they’re published. (Participant 248)

#### 3.7.3. Experience of study process

Some participants commented on their approach to answering questions or how they experienced the delivery of the study:

One personal complication for me was in separating my feelings out to focus on just one loss. (Participant 145)

I feel very professionally and compassionately held by you in this process even by the way the questionnaire was phrased. (Participant 147)

#### 3.7.4. Coping strategy after questionnaires

In the initial contact with the researcher, participants were advised to engage in coping strategies appropriate to them following completion of the questionnaires. Occasionally, participants described methods they had used to support themselves during and after taking part:

I waited to complete it until I knew I could talk to someone about things. (Participant 120)

## 4. Discussion

Given that some concerns have been expressed regarding the participation of vulnerable groups in research, we aimed to explore how bereaved individuals experience online research participation about grief. In this study, over one-third of participants chose to respond to an email they were sent checking on their well-being the day after participation. Of these responses, the majority included a comment on the emotional impact of completing the questionnaires, most of which indicated that they felt okay doing this, or experienced some negative emotions that quickly resolved. Responses also included comments suggesting a number of possible benefits to taking part. We will discuss recommendations for studies that include potentially vulnerable populations.

The present findings suggest that completing online questionnaires about grief was manageable for individuals who had lost someone and that any negative emotional reactions surrounding the questionnaires were temporary. These results are in line with previous work that suggested that research participation in traumatic stress studies is well tolerated (Newman & Kaloupek, 2004). Participants in this study included individuals who could be considered particularly vulnerable, namely those in the first few months after losing a significant other, those who had lost a child, and those bereaved through violent means. Group comparisons to examine whether these groups require specific recommendations were not within the scope of the present study, but would be helpful in future studies. Only 2% of participants in this study did not explicitly indicate that negative emotional effects had resolved within 24 hours. Indeed, one-third of participants chose to respond to the email by sharing substantial additional information about their loss and reactions, further to the information that they had already shared through the standard questionnaires. This suggests that they felt emotionally contained by the process rather than being threatened or upset by it. This is supported by the fact that only six participants accepted the offer of a further telephone call to discuss difficult aspects of participation. This would appear to indicate that researchers need not be concerned that offering this additional support will lead to a significant increase in research time. Such findings are in line with previous studies examining face-to-face and postal methods of research participation with vulnerable groups (Cook & Bosley, 1995; Dyregrov, 2004; Jaffe, DiLillo, Hoffman, Haikalis, & Dykstra, 2015) and appear to indicate that the online method of participation does not influence this outcome.

Participants mentioned a range of positive aspects to taking part, including being interested in the study topic and appreciating the chance to contribute to research and help others. Participants also described valuing the opportunity to think about the loss and put their thoughts and feelings into words; for some participants, the questionnaires prompted helpful self-reflection, which sometimes led to new realizations around their grief, their use of coping strategies, or changes in these over time. Given these unanticipated consequences, future studies may wish to use the themes derived from the email content to design a structure for measuring the impact of research in a more quantitative way. While it is encouraging that some participants described such benefits during an emotionally difficult time, we should bear in mind the possible research implications. Although not common, it appears that the act of measuring someone’s experience of distress may have had some degree of therapeutic impact; the so-called ‘measurement reactivity’ effect (French & Sutton, 2010). Researchers may therefore need to consider this when designing longitudinal studies, for example by opting for fewer or less frequent measurement points to try to minimize this effect. Further experimentally controlled research to examine the extent of this effect is warranted. Comparing participants who complete grief questionnaires to those who complete an equivalent set of neutral questionnaires could be one method to test this.

Dyregrov (2004) suggested guidelines for conducting research with vulnerable populations, such as emphasizing informed consent, providing flexibility on where and when to participate, and including additional follow-up. The fact that participants in the present study described feeling pleased to be taking part, positive experiences of the process, and appreciation of the check-in email suggests that the present methods were successful in following these guidelines. For researchers planning studies similar to the present one, we offer the following recommendations, which are based on our experiences of conducting this study and the email responses received:

1. Consider online recruitment and participation if possible. There are obvious practical advantages (wider reach, simultaneous participation, no travel costs, etc.), but also potential ethical ones, in that people can participate at any time, in a comfortable location, and can pause or stop without the pressures of being in a clinical or research environment. This approach also means that participants are not recruited in clinical settings, which may be difficult (e.g. if it is where the deceased died) or lead to coercion (i.e. if they feel obliged to help the service in which the deceased was treated), meaning that there is perhaps more opportunity to fully consider their participation before giving informed consent.
2. Offer a general set of well-being guidelines at the beginning of participation. We suggest that participants are encouraged to choose when and where to complete questionnaires, to take breaks if needed, and to engage in coping strategies appropriate to them, for example planning a relaxing or distracting activity for afterwards.
3. Send a check-in email 24 hours after their participation (this can be automated if the study involves large samples). As outlined in the present study, this step was very much appreciated by participants. We think it is important in providing an opportunity to explain more or express things that were not directly covered within the study. In addition, it may allow participants to engage with the questionnaires more fully, knowing that they have the opportunity for further support if necessary.

This study has a number of strengths and limitations. One strength is that as participants were not asked to respond to the check-in email, or to reply to any direct questions, their responses may have been more able to capture an authentic sense of their reactions. However, it is possible that more data could be obtained by taking an approach of direct questioning, for example about the emotional impact of the questionnaires or the experience of participation overall. This would be an important next step in verifying the present findings. People who responded to the email were on average older than those who did not, but otherwise there were no differences in demographics, loss characteristics, or psychological functioning. This could indicate that the themes extracted from the responder group may apply to the non-responders or potentially to participants in similar studies, although not having a control group means that we cannot rule out the possibility that those who did not reply had other, including more negative, experiences of participation.

These findings suggest that participants in an online study about grief found it manageable and acceptable. Although negative emotional reactions were commonly described as arising from reflecting on their grief, these were largely reported to be temporary. In addition, several benefits were reported. We suggest that the procedures followed to support well-being contributed to positive experiences of participation, and we recommend these for future online studies. These findings could allay clinical concerns about conducting this type of research with vulnerable populations, as well as raising methodological questions about the possible impact of measurement.
