# Supplementary material for: Conducting ethical internet-based research with vulnerable populations: a qualitative study of bereaved participants’ experiences of online questionnaires
Source: Eur J Psychotraumatol. 2018 Aug 21;9(Suppl 1):1506231. doi: 10.1080/20008198.2018.1506231 (PMC6104613; doi:10.1080/20008198.2018.1506231)
Supplement: Supplemental Material [file ZEPT_A_1506231_SM6662.docx]

Dear [-]

I wanted to write and thank you for completing the questionnaires yesterday and to say how sorry I am for your loss. Being a part of The Oxford Grief Study is obviously a membership that everyone would rather not have so I appreciate you giving your time to reflect on such a difficult and sensitive subject.

Given the nature of the material being thought about and considered I wanted to check in that you were feeling ok today. It can often be very emotional turning towards grief and it is important to me that you feel safe throughout the research process. If any difficult emotions have come up today I wanted to remind you that this is completely normal and understandable and should subside to pre-research levels with time.

However, if you feel you would benefit from a telephone chat over the next few days to discuss any aspects that you found difficult then please do let me know.

With warm wishes,
